# Chaihulonggumulitang Shows Psycho-cardiology Therapeutic Effects on Acute Myocardial Infarction by Enhancing Bone Marrow Mesenchymal Stem Cells Mobilization

**DOI:** 10.1038/s41598-018-21789-w

**Published:** 2018-02-27

**Authors:** Chao Wang, Hongsen Du, Jiqiu Hou, Shasha Yan, Jingjing Yang, Yun Wang, Xiujing Zhang, Lili Zhu, Haibin Zhao

**Affiliations:** 1 Graduate School, Beijing University of Chinese Medicine, Beijng, (100029) China; 2 The Third Affiliate Hospital of Beijing University of Chinese Medicine, Beijing, (100029) China

## Abstract

Ischemic myocardium initiates the mobilization and homing of bone marrow mesenchymal stem cells (BM-MSCs) to promote myocardial regeneration after acute myocardial infarction (AMI). Inflammation caused by necrotic cardiomyocytes induce major pathological changes (cardiac remodeling and myocardial apoptosis) as well as anxiety disorder. This process may be inhibited by the differentiation and paracrine effects of BM-MSCs. However, the spontaneous mobilization of BMSCs is insufficient to prevent this effect. Given the anti-inflammatory effects of BM-MSCs, ventricular remodeling and anxiety following AMI, methods focused on enhancing BMSCs mobilization are promising. BFG is a classical traditional Chinese prescription medicine and has been proved effective in treating AMI and reducing anxiety, but the potential mechanism of its function remains unknown. In the present study, we explored the effects of Chaihulonggumulitang (BFG) on AMI and anxiety *in vivo and in vitro*. We also tested its effects in promoting BMSCs mobilization and alleviating inflammation. Our data showed that the classical Chinese prescription BFG promoted BM-MSCs mobilization, inhibited inflammatory response, and improved heart damage and anxiety developed from AMI. Thus, we provided an underlying mechanism of BFG function in psycho-cardiology conditions such as AMI.

## Introduction

Cardiovascular disease is a leading cause of mortality and morbidity worldwide. Acute myocardial infarction (AMI) associated fatal conditions, such as heart failure and sudden cardiac death are an enormous psychological and financial burden on patients and society^1^. The inflammatory response caused by injured myocardium initiate ventricular remodeling and myocardial apoptosis, leading to dysfunctional myocardium^2^. Self-regenerative capability of cardiomyocytes is limited. Although the mortality is lower due to medical advances and surgery, the inevitable progressive disease carries significant morbidity is merely transient delayed^3^.

Anxiety is a risk factor for cardiovascular disease and is frequently associated with inflammation induced by the injured myocardium^4,5^. With an incidence rate of 25% in acute coronary syndrome (ACS) patients, anxiety is regarded as a chronic condition that can persist for up to 1 year in half of the post-ACS patients. Enhanced anxiety in 3 months following myocardial infarction predicts adverse cardiac events and mortality^6,7^. Given its high morbidity and association with poor cardiac health, it is clear that patients suffering from ACS require coordination of mental health care and cardiac treatment, also known as psycho-cardiology therapy. However, the response to conventional anti-anxiety methods has achieved limited success in ACS patients. Thus, there is a pressing need for improvement in psycho-cardiology treatments in response to AMI^8^.

Cellular therapy has emerged as a leading approach for cardiac regeneration. Bone marrow mesenchymal stem cells (BM-MSCs), with their differentiation and paracrine capability, are attractive candidates for stem cell-based therapy. The stress signals released by necrotic myocardium mobilize BM-MSCs to migrate from bone marrow to circulating blood, from where they home to the injured region^9,10^. Once aggregated at the injured site, BM-MSCs alleviate inflammatory response, stimulate angiogenesis, and restore contractile function by their self-differentiation and paracrine properties. As a result, scarred or dysfunctional myocardial tissue is replaced with contractile and perfused tissue^11–13^. However, the spontaneous mobilization of BM-MSCs is not insufficient to achieve desired protective effects^14^. The success rate of BM-MSCs transplantation is quite low due to the harsh microenvironment at the injured region. The invasive approaches, such as intracoronary and intravenous infusion, may also limit the application of BM-MSCs transplantation^10^.

Given the anti-inflammatory effects of BM-MSCs and their protective role in ventricular remodeling and anxiety following AMI, methods that focused on enhancing BM-MSCs mobilization are promising. As a complementary and an alternative therapy, traditional Chinese medicine (TCM) might improve the pathological features of AMI through multiple pathways^15–17^. Chaihulonggumulitang (Bupleurum Falcatum Dragon Bone and Oyster Shell Granule, BFG) is a traditional Chinese prescription derived from the classic *Treatise on Febrile and Miscellaneous Diseases*. It contains Radix bupleuri, Dragon bone, oyster shell, Radix scutellariae, Ramulus cinnamomi, Minium, Pinellia ternate, ginger, Rheum officinale, Poria cocos, Radix ginseng and Fructus zizyphi. BFG is effective in the treatment of cardiovascular disease as well as anxiety disorder, which fulfills the philosophy of psycho-cardiology^18–20^. However, the role of BFG in psycho-cardiology treatment of AMI and its definitive mechanism of action is not known. We hypothesized that BFG may act by enhancing BM-MSCs mobilization to inhibit the inflammatory response, and improve heart damage and anxiety developing from AMI.

## Materials and Methods

### Animals

Male Sprague-Dawley (SD) rats were purchased from Beijing Vital River Laboratory Animal Technology Co. Ltd. (License No. SCXK (Beijing)2016–0006). Rats were fed a standard laboratory diet and kept in a room with controlled temperature (22 ± 1 °C), relative humidity (65–70%), and a 12:12 light/dark cycle. All animal procedures were approved and carried out following the guidelines of the Institutional Animal Care and Use Committee of University of Chinese Medicine, Beijing, China. Every effort was made to minimize the suffering and number of rats used in this study.

### Drugs

BFG was purchased from Beijing Pharmaceutical Co. Ltd. One dose of BFG consisted of: Radix bupleuri 12 grams (g), Dragon bone 15 g, oyster shell 15 g, Radix scutellariae 9 g, Ramulus cinnamomic 9 g, Nacre (instead of Minium) 15 g, Pinellia ternate 9 g, ginger 9 g, Rheum officinale 9 g, Poria cocos 15 g, Radix ginseng 9 g and Fructus zizyphi 10 g. One dose of BGF was dissolved in 100 ml distilled water to prepare the BFG solution. Our preliminary experimental results showed that the optimal dosage of BFG solution for rats was 6 times of adult dosage. The dosage of BFG solution for rats was adjusted to the body weight by 1 ml (BFG solution)/100 g (body weight) qd.

### *In vivo* study

#### Establishment of the Myocardial Infarction (MI) Rat Model

Coronary artery ligation was used to establish the MI rat model. Rats weighting 250 ± 10 g were anesthetized with 1% pentobarbital sodium (0.5 ml/100 g) intraperitoneally and artificially ventilated with a small animal ventilator with a tidal volume of 1 ml at a rate of 100 cycles/min. Heart was exposed via a small retractor after thoracotomy was performed at the left third intercostal space. The left anterior descending coronary artery was ligated directly under the origin 2–3 mm of left atrial appendage by a 5–0 suture. 2–0 sutures were used to close thorax and skin. For the sham surgery, similar surgery was operated except that no ligature was placed. All operations were conducted under sterile conditions. Q wave in electrocardiogram the day after surgery was used to confirm the successful ligation.

#### Design and allocation

72 rats were randomly assigned into three groups (n = 24 per group): the sham group, the model group and the MI + BFG group. Rats in each group were further divided into three subgroups (n = 8) according to the duration of administration of BFG (3 days, 7 days and 14 days). Rats in the MI + BFG group was given with a dosage of 1 ml (BFG solution)/100 g (body weight) of BFG solution by gavage once a day, while those in the sham and the model group received the same volume of distilled water. At the end of administration, rats underwent behavioral tests and were subsequently anaesthetized for echocardiography. After echocardiography, blood samples were collect and analyzed by flow cytometry assay and enzyme linked immunosorbent assay (ELISA). Heart and brain tissues were collected for pathological staining, immunohistochemistry, and real-time quantitative RT-PCR.

#### Echocardiography

Echocardiography was conducted to evaluate the function and structure of left ventricle in each group at different timepoints. Rats were fixed on their backs with fur shaved after anesthesia with 1% pentobarbital sodium (0.5 ml/100 g) intraperitoneally. Left ventricular ejection fraction (LVEF) and left ventricular fractional shortening (LVFS) were measured to evaluate heart function. The left ventricular end-diastolic inner diameter (LViDd), left ventricular end-systolic inner diameter (LViDs), left ventricular end-diastolic volume (LVEDV), left ventricular end-systolic volume (LVESV), left ventricular anterior wall end-diastolic thickness (LVAWTd), left ventricular posterior wall end-diastolic thickness (LVPWTd) and interventricular septum end-diastolic thickness (IVSTd) were measured to evaluate the heart structure. Echocardiography was operated by a technician who was blinded to the grouping allocation.

#### Behavioral tests

Behavioral tests were conducted to measure anxiety-like behavior after AMI. Rats were brought to testing room approximately 30 min before the test and all behavioral tests were performed in a double-blinded manner. Behavior was videotaped by the camera hanging over the test apparatus.

Open-field test (OFT) test apparatus is a square wooden case (100 cm*100 cm*60 cm) with the floor divided into 25 identical squares (20 cm*20 cm) with white stripes. The test was operated in a dark and quiet room. A single rat was placed in the center of the case and allowed to explore for 3 min. The number of squares crossed by rats was recorded as a horizontal movement score (with all four paws crossing the line, each line cross was scored as 1 point), while the rearing behavior was recorded as a vertical movement score (each rearing was scored as 1 point). The arena was wiped cleaned with 75% alcohol.

Elevated plus-maze test (EPM) apparatus consists of two open arms (50 cm*10 cm) and two closed arms (50 cm*10 cm, surrounded by 40 cm walls) that originate from a common central platform(10 cm*10 cm). EPM was elevated to a height of 50 cm above the floor in a dark and quiet room. A single rat was placed on the platform facing the open arms and allowed to explore for 5 min. The proportion of time spent in the open arms (OAT, = the time spent in the open arms/5 min) and the proportion of numbers of entries in the open arms (OAN, = the number of entries into the open arms/the numbers of entries into the open arms and closed arms) were calculated. The maze was cleaned with 75% alcohol.

#### Pathological staining

Histopathological changes in heart after AMI were evaluated by staining. Rats were anesthetized with 1% pentobarbital sodium (0.5 ml/100 g) intraperitoneally and perfusion-fixed. Heart samples were isolated and embedded in paraffin after fixation in 4% paraformaldehyde for 72 h. Tissues were sectioned at a thickness of 4μm and stained with haematoxylin and eosin pathological (H&E) staining and Masson trichrome staining. Four fields of each slice were chosen for quantification. Image J software was used to calculate the percentage of myocardial infarction area and the percentage of collagen fiber accounting for the total infarction area.

#### Flow cytometry assay

Mobilization of BM-MSCs *in vivo* was measured by flow cytometry assay. Antibodies used in flow cytometry assay were purchased from Beijing Biosynthesis Biotechnology Co. Ltd. The surface molecules markers CD90, CD105 of BM-MSCs, and adhesion-related antigens CD54 and CD106 were measured. Briefly, Rats were anesthetized as mentioned above. Bone marrow of thigh bone was flushed out with phosphate buffer (PBS) and treated with erythrocyte lysis buffer to remove erythrocytes. Blood sample were collected from abdominal aorta and placed in an anticoagulant tube containing EDTA and treated with erythrocyte lysis buffer to remove erythrocytes. After washing with PBS 3 times, bone marrow cells and blood samples were then resuspended in PBS. Bone marrow cells were incubated with anti-CD90-PE, anti-CD105-APC, anti-CD54-APC and anti-CD106-PE while blood samples were incubated with anti-CD90-APC, anti-CD105-PE, anti-CD54-PE and anti-CD106-APC for 30 min. Unconjugated antibody was washed off and cells were resuspended in PBS for testing. Approximately 1 × 10^6^ bone marrow or blood cells were placed into the flow cytometry chamber (BD Pharmingen, San Diego, CA, USA) and 1 × 10^4^ cells were counted in each sample to calculate the percentages of positive cells.

#### Immunohistochemistry

Immunohistochemistry was used to evaluate the homing of BM-MSCs in the marginal zone of myocardial infarction and the glutamic acid decarboxylase 67 (GAD67) content in brain. The paraffin sections of heart and brain tissue were processed as mentioned previously. Anti-CD105 (biorbyt, China) was incubated with heart sections, while anti-GAD67 (abcam, USA) was incubated with brain sections after routine dewaxing, hydration and antigen retrieval. Results were analyzed with Image-pro-plus software. Eight fields of each slice were randomly chosen and the integrated optical density (IOD) was calculated.

#### Enzyme linked immunosorbent assay (ELISA)

ELISA was used to detect inflammation marker nuclear factor-κB (NF-κB), tumor necrosis factor-α (TNF-α) and anxiety-related hormones adrenaline, and stem cell factor (SCF) in peripheral serum after AMI according to manufacturer’s instructions. ELISA kits were purchased from Beijing Biosynthesis Biotechnology Co. Ltd.

#### Real-time Quantitative RT-PCR

Real-time Quantitative RT-PCR was used to measure the expression of neurotransmitter receptor mRNA associated with anxiety. Total RNA of brain hippocampus was extracted by E.Z.N.A. Total RNA Kit I (Omega, USA). Reverse transcription was performed with TRUEscript One Step qRT-PCR Kit (Aidlab, China). PCR reactions were performed with SYBR green master mix (ABI, Hercules, CA). The specific primers pairs are as follows: NMDAR1 mRNA forward 5′-AATGACCCCAGGCTCAGAAAC-3′ and reverse 5′-TGAAGCCTCAAACTCCAGCAC-3′(222 bp); GABAAR mRNA forward 5′-GAGAGTCAGTACCAGCAAGAAC-3′ and reverse 5′-AGAACACGAAGGCATAGCAC-3′(150 bp); Rats GAPDH endogenous reference genes primers were purchased from the Sangon Biotech. Melting curve analysis was used to verify the accuracy of the amplicon. The specificity of reaction was assessed by a negative control without cDNA run with every PCR. Data was analyzed with Bio-Rad CFX Manager software. PCR efficiency for all primer sets ranged from 90% to 110%. Data were calculated as the change in cycle threshold (ΔC_T_) for target gene relative to the ΔC_T_ for GAPDH (control gene) according to procedures of Muller’s *et al*.^21^.

### *In vitro* study

#### Cell isolation and culture

The whole bone marrow adherent method was used for isolating primary BM-MSCs. Male SD rats weighting 150 ± 10 g were chosen. Bone marrow of tibias and femurs was extracted thoroughly with a 10 ml syringe using low-glucose Dulbecco’s modified Eagle’s medium (L-DMEM, HyClone, USA) under sterile conditions. Marrow cells suspension was filtered by a 200-mesh sieve, centrifuged, and cultured in L-DMEM supplemented with 15% (v/v) fetal bovine serum (FBS, Gibco, USA), 100U/ml penicillin, and 100 μg/ml streptomycin (HyClone, USA) (hereafter referred to as a complete medium) at 37 °C in a 5% CO_2_ incubator for 48 h. Half of the medium was changed 48 h later. Thereafter, medium was changed every three days. Upon reaching 80% confluency, cells were trypsinized with 0.25% trypsin (HyClone, USA) for passage. Cells of passage three were used for the subsequent experiments.

#### Preparation of medicated serum

Rats weighing 250 ± 10 g were chosen and randomly divided into BFG group (BFG serum) and control group (control serum). Rats in BFG groups were treated intragastrically with a BFG solution at a dosage of 1 ml/100 g/day (n = 6) while the control group received the same volume of sterile water (n = 6). Treatments were administered for five consecutive days. The abdominal aortic blood samples were collected under sterile conditions one hour after the final intragastric administration. Blood sample was aseptically packed and centrifuged at 12,000 g for 15 min. Serum was inactivated at 56 °C for 30 min, filter with a 0.22 μm filter membrane, stored at −80 °C.

#### Group design and preparation of cells

BM-MSCs of passage three were divided in three groups: BFG group, control group and blank group. BM-MSCs were seeded into 6-wells plates (2 × 10^5^ cells per well) and cultured in complete medium. Cells in BFG groups were treated with complete medium containing 5% BFG serum, while those in control groups were treated with complete medium containing 5% control serum. Cells cultured with complete medium served as the blank group. Migratory ability of BM-MSCs were determined using the tablet scratch assay and Transwell assay 24 h after medicine administration.

#### Tablet scratch assay

BM-MSCs were cultured in 6-wells plates (2 × 10^5^ cells per well) in complete medium for 24 h. The medium was then changed to a serum-free medium to inhibit proliferation for 12 h. A cell-free strip was generated by scratching the cellular monolayer with a pipette tip (200 μl). The distance was recorded as 0 h. BFG serum (5%) or control serum (5%) was added to the cells. Distance of strips in each group was recorded after 18 h incubation. Scratch shortening rate ( = distance in 0h-distance in 18 h/distance in 0 h) was calculated in each group.

#### Transwell assay

The Transwell membrane system (corning, USA) contains an 8μm pore polycarbonate membrane in a 24-well culture plate. Briefly, cells in different groups were treated for 24 h. Treated cells were resuspended in medium with 0.2%FBS before transferring them to the upper chamber of a Transwell system (2 × 10^4^ cells per well). 600 μl of medium containing 20% FBS was added to each lower chamber. Cells on membranes of Transwell insert were fixed with 4% paraformaldehyde for 30 minutes after 6 h and 12 h incubation. Non-migrating cells on the upper surface of the well were detached from the membrane. The migrated cells on the lower surface of the membrane were stained with a 0.2% crystal violet solution. Finally, six random fields (100×) of each group were randomly chose for cell counting.

### Statistical analysis

Statistical Package for Social Sciences (SPSS) for windows (version 22.0) was used to analyze the data. Data was showed as mean ± standard deviation(SD) and analyzed by one-way analysis of variance (ANOVA). Fisher’s least significant difference (LSD) test was used to conduct the multiple comparisons. A value of P < 0.05 was considered statistically significant.

## Results

### BFG attenuated heart function injury and ventricular reconstruction in myocardial infarcted rats

**Figure 1:**
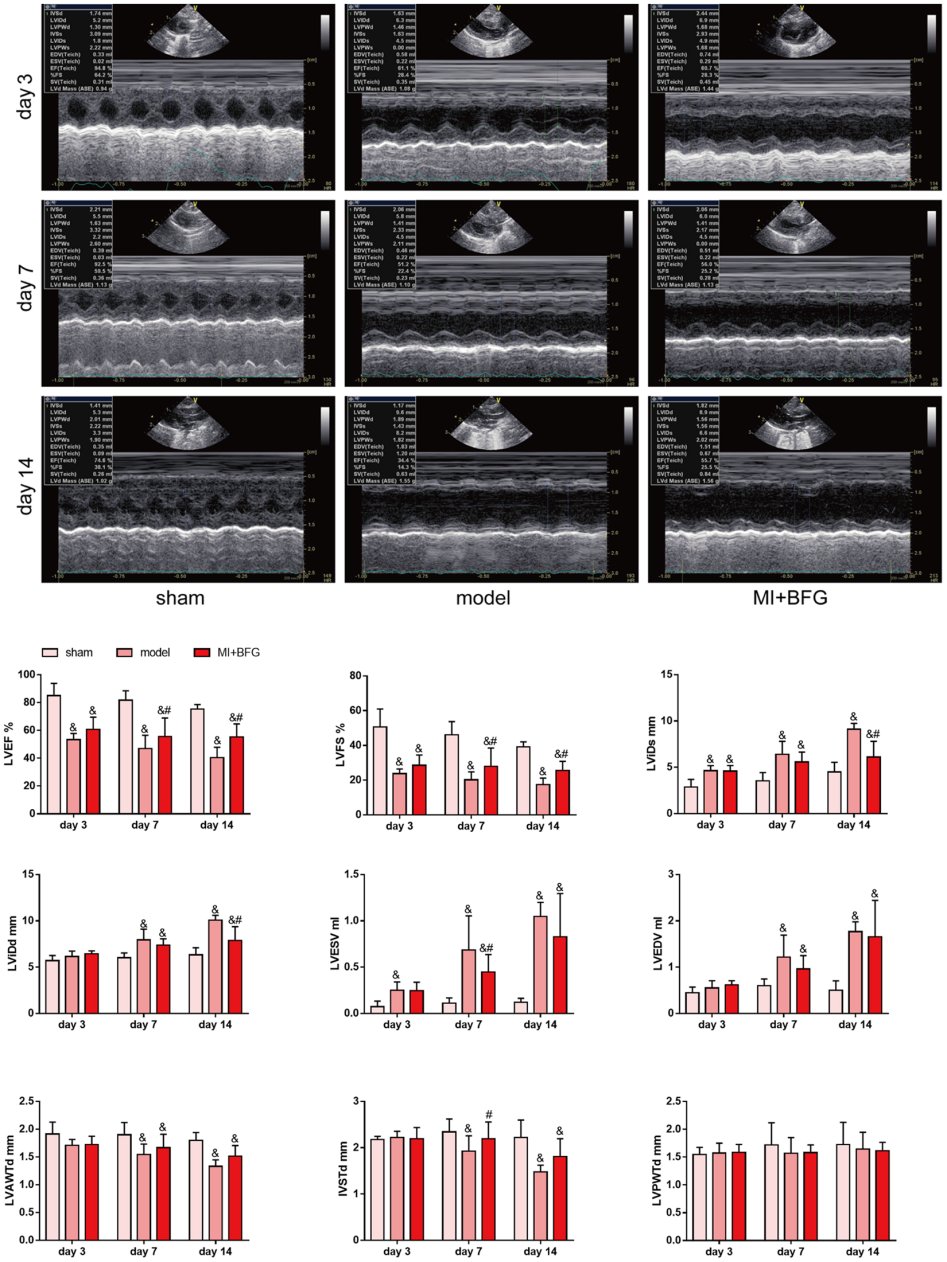
Changes of ventricular reconstruction hemodynamics and cardiac function parameters (n = 8). Values are expressed as mean ± SD. LVEF: Left ventricular ejection fraction; LVFS: Left ventricular fractional shortening; LViDs: left ventricular end-systolic inner diameter; LViDd: left ventricular end-diastolic inner diameter; LVESV: left ventricular end-systolic volume; LVEDV:left ventricular end-diastolic volume; LVAWTd: Left ventricular anterior wall end-diastolic thickness; LVPWTd: left ventricular posterior wall end-diastolic thickness; IVSTd: interventricular septum end-diastolic thickness. BFG: Chaihulonggumulitang (*Bupleurum falcatum plus Dragon Bone and Oyster Shell Granules)*. MI: myocardial infarction. Sham group: rats with sham surgery. model group: myocardial infarcted rats. MI + BFG group: acute myocardial infarction rats with administration of BFG. ^&^P < 0.05, compared with the sham group at the same time-points; ^#^P < 0.05, compared with the model group at the same time-points.

As shown in Fig. 1, the LVEF and LVFS decreased over time after ligation surgery in the model group and were statistically different from the sham group (which remained stable) on day 3, 7, and 14 (P < 0.05). In contrast, the LVEF and LVFS in MI + BFG group significantly increased when compared to the model group on day 7 and 14 (P < 0.05). Thus, BFG overcame the inhibitory effects of MI on LVEF and LVFS (Fig. 1).

The left ventricle expanded in the model group and in the MI + BFG group in comparison to the sham group after ligation surgery (Fig. 1). LViDd and LVEDV in the model and MI + BFG group increased significantly on day 7 and 14 compared to the sham group (P < 0.05). LViDs and LVESV in the model and MI + BFG group also increased significantly on day 3, 7 and 14 in comparison to the sham group (P < 0.05). Administration of BFG decreased the LViDd and LViDs on day 14 and the LVESV on day 7 (P < 0.05) but did not affect LVEDV (P > 0.05).

LVAWTd and IVSTd in the model group and in the MI + BFG group were lower than those in the sham group on day 7 and 14 (P < 0.05). Compared to the model group, IVSTd in the MI + BFG group increased on day 7 (P < 0.05) but no difference was found in LVPWTd levels among these groups (Fig. 1).

### BFG alleviated heart tissue histologic injury in myocardial infarcted rats

**Figure 2:**
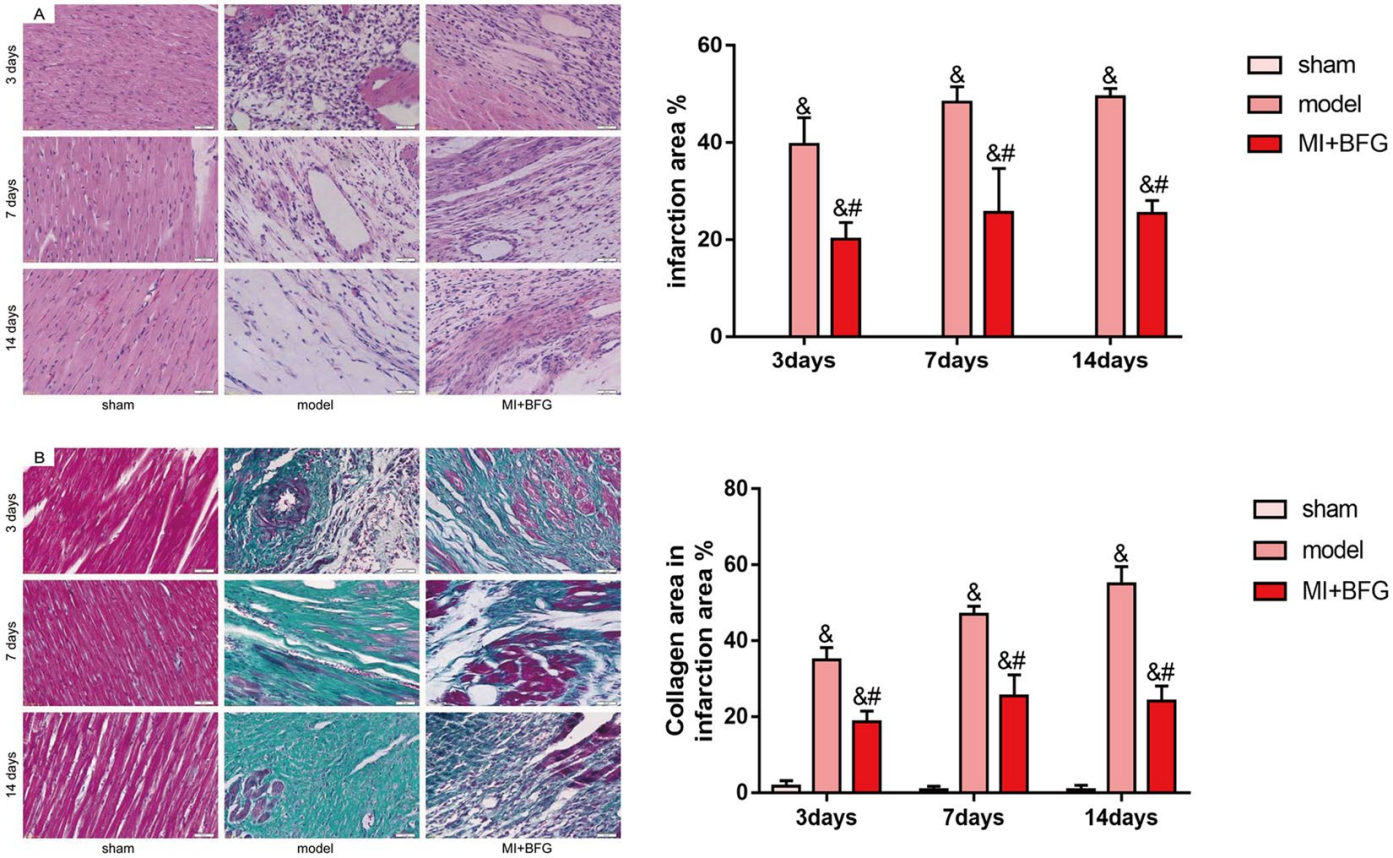
Myocardial histopathological changes in myocardial infarcted rats (×400 magnification, n = 8). (**A**) HE staining; (**B**) Masson staining. BFG: Chaihulonggumulitang (*Bupleurum falcatum plus Dragon Bone and Oyster Shell Granules)*. MI: myocardial infarction. Sham group: rats with sham surgery. Model group: myocardial infarcted rats. MI + BFG group: acute myocardial infarction rats with administration of BFG. ^&^P < 0.05, compared with the sham group at the same time-points; ^#^P < 0.05, compared with the model group at the same time-points.

H&E staining showed that myocardial fibers in the sham group were orderly arranged with regular nuclei and uniform cytoplasmic staining at all timepoints tested. Conversely, those in the model group showed a disordered arrangement of myocardial fibers with a wide range of necrosis and numerous infiltrating neutrophils, which deteriorated from 3 to 14 days. These effects were alleviated in MI + BFG group. Masson staining showed that myocardial collagen fibers increased in the model group with fibrous scar formation. There was some degree of alleviation in the MI + BFG group. Quantitative analysis showed the model group had a larger infarction size and collagen area than the sham group and these changes were alleviated by BFG administration (P < 0.05) (Fig. 2).

### BFG promoted mobilization of BM-MSCs in myocardial infarcted rats

**Figure 3:**
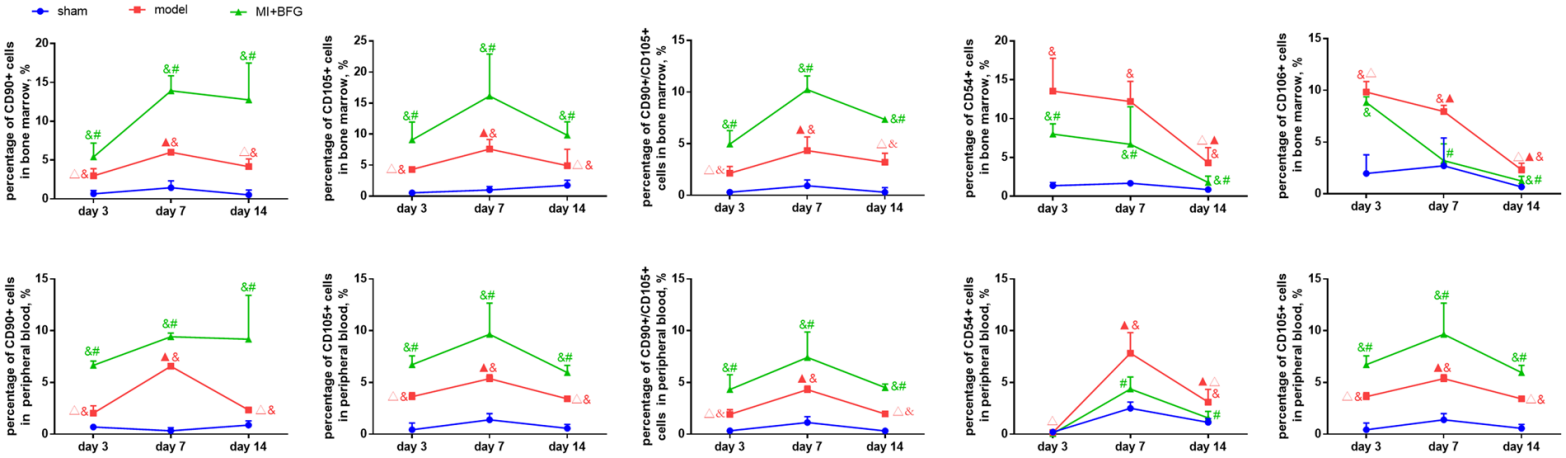
Mobilization of bone marrow mesenchymal stem cells in myocardial infarcted rats (n = 8). Values are expressed as mean ± SD. BFG: Chaihulonggumulitang (*Bupleurum falcatum plus Dragon Bone and Oyster Shell Granules)*. MI: myocardial infarction. Sham group: rats with sham surgery. Model group: myocardial infarcted rats. MI + BFG group: acute myocardial infarction rats with administration of BFG. ^&^P < 0.05, compared with the sham group at the same time-points; ^#^P < 0.05, compared with the model group at the same time-points; ^Δ^P < 0.05, compared with the model group on day 7; ^▲^P < 0.05, compared with the model group in day 3.

Number of CD90 or CD105 positive cells in bone marrow and peripheral blood in the model group was higher than that in sham group (P < 0.05). Peak values were observed on day 7 after the ligation surgery in model group and was statistically different when compared to day 3 and 14 (P < 0.05). Administration of BFG increased the number of CD90 or CD105 positive cells in bone marrow and blood at all time-points, when compared to the model group (P < 0.05) (Fig. 3). The number of cells that co-expressed CD90 and CD105 was a bit lower than that only expressed CD90 or CD105 in both bone marrow and blood. All three type cells shared the same changing trend in quantity (Fig. 3).

Number of CD54 or CD106 positive cells in bone marrow in the model group showed a reverse trend. They peaked on day 3 after the ligation surgery and declined afterwards. Those in MI + BFG group showed the same changing trend but lower values than those in model group on day 7 and 14 (P < 0.05). The number of CD54 or CD106 positive cells in peripheral blood in the model group increased significantly and peaked on day 7, and was different from the sham group on day 7 and 14 (P < 0.05). The number of these cells declined in MI + BFG group when compared to the model group on day 7 and 14 (P < 0.05) (Fig. 3).

**Figure 4:**
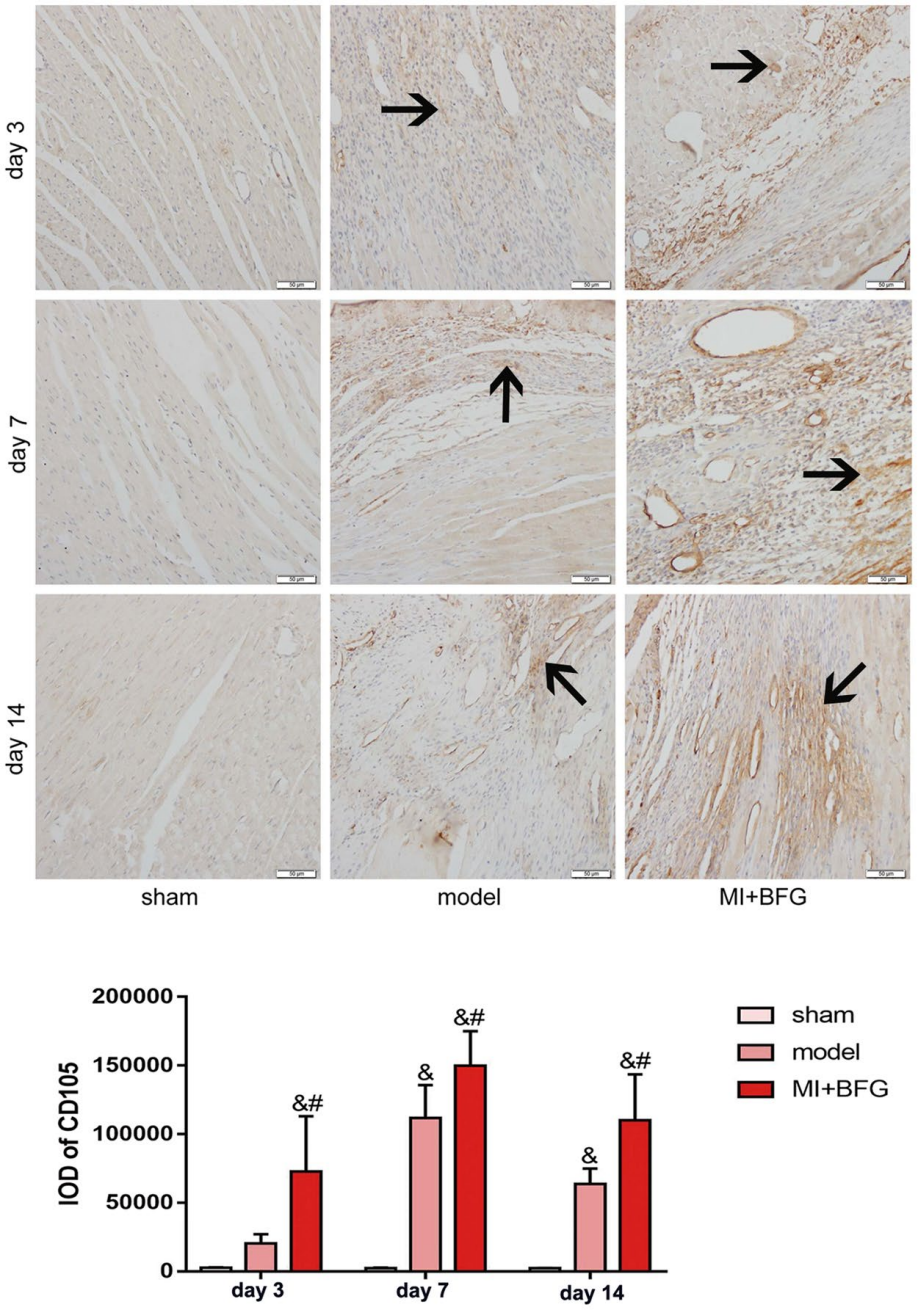
CD105 expression in the marginal zone of myocardial infarcted rats (n = 8) (Immunohistochemistry, ×200 magnification). Values are expressed as mean ± SD. IOD: integrated optical density. BFG: Chaihulonggumulitang (*Bupleurum falcatum plus Dragon Bone and Oyster Shell Granules*). MI: myocardial infarction. Sham group: rats with sham surgery. Model group: myocardial infarcted rats. MI + BFG group: acute myocardial infarction rats with administration of BFG. ^&^P < 0.05, compared with the sham group at the same time-points; ^#^P < 0.05, compared with the model group at the same time-points.

In comparison to the sham group, CD105 expression in marginal zone of AMI was higher in the model group on day 7 and 14 (P < 0.05). CD105 expression was further elevated in MI + BFG group compared to the model group on day 3, 7 and 14 (P < 0.05) (Fig. 4).

**Figure 5:**
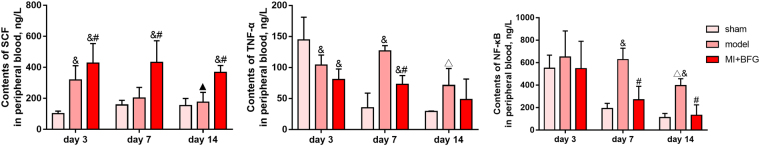
Contents of SCF, NF-κB and TNF-α in peripheral blood of myocardial infarcted rats (n = 8). Values are expressed as mean ± SD. SCF: stem cell factor; TNF-α: tumor necrosis factor-α; NF-κB: nuclear factor-κB. BFG: Chaihulonggumulitang (*Bupleurum falcatum plus Dragon Bone and Oyster Shell Granules*). MI: myocardial infarction. Sham group: rats with sham surgery. Model group: myocardial infarcted rats. MI + BFG group: acute myocardial infarction rats with administration of BFG. ^&^P < 0.05, compared with the sham group at the same time-points; ^#^P < 0.05, compared with the model group at the same time-points; ^△^P < 0.05, compared with the model group on day 7; ^▲^P < 0.05, compared with the model group in day 3.

The SCF expression was maintained at low level in the sham group at all timepoints. It peaked on day 3 after AMI in the model group but dropped down to about the same level as that in sham group on day 14. The MI + BFG group showed a higher level of SCF in peripheral blood when compared to the model group at all timepoints (p < 0.05) (Fig. 5).

### BFG alleviated the inflammatory response in myocardial infarcted Rats

As shown in Fig. 5, NF-κB and TNF-α level in peripheral blood gradually descended after the ligation surgery in the sham group while those in model group remained high. BFG administration reduced blood NF-κB levels on day 7 and 14 when compared with the model group (p < 0.05). TNF-α content in the MI + BFG group also declined on day 7 in comparison to the model group (p < 0.05) (Fig. 5).

### BFG improved anxiety degree in myocardial infarcted rats

**Figure 6:**
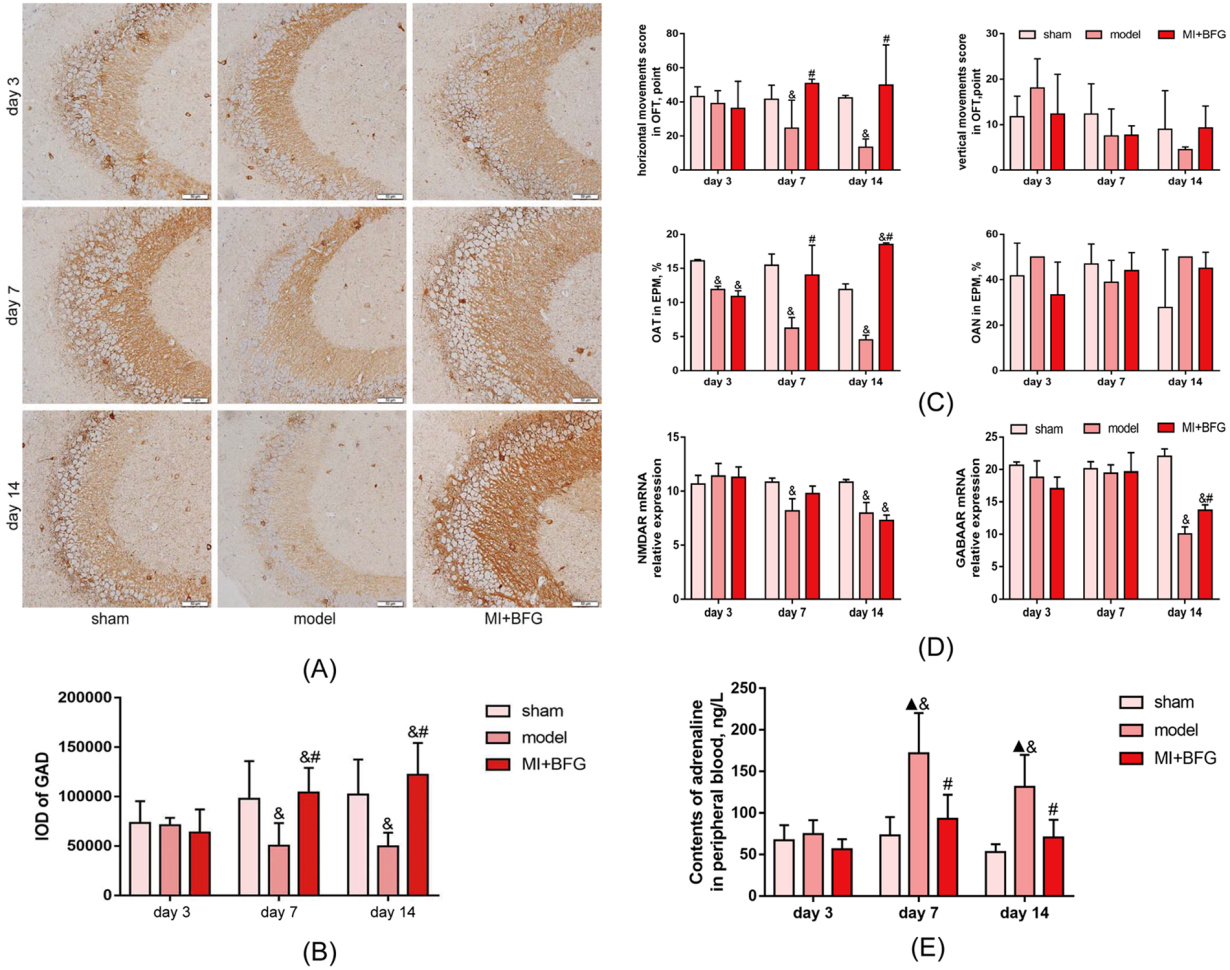
Anxiety degree of myocardial infarcted rats (n = 8). (**A** and **B**) Level of GAD67 expression in hippocampus of myocardial infarcted rats (Immunohistochemistry, × 200 magnification). (**C**) Behavioral tests of myocardial infarcted rats. (**D**) Relative expression of glutamate receptor NMDAR mRNA and γ-aminobutyric acid receptor GABAAR mRNA in hippocampus of myocardial infarcted rats. (**E**) Contents of adrenaline in peripheral blood of myocardial infarcted rats. Values are expressed as mean ± SD. IOD: integrated optical density. GAD67: glutamic acid decarboxylase 67. OFT: Open-field test; OAT: The proportion of time spent in the open arms; OAN: the proportion of numbers of entries into the open arms. EPM: Elevated plus-maze test. NMDAR: N-methyl-D-aspartate receptor; GABAAR: γ-aminobutyric acid A receptor. BFG: Chaihulonggumulitang (*Bupleurum falcatum plus Dragon Bone and Oyster Shell Granules*). MI: myocardial infarction. Sham group: rats with sham surgery. Model group: myocardial infarcted rats. MI + BFG group: acute myocardial infarction rats with administration of BFG. ^&^P < 0.05, compared with the sham group at the same time-points; ^#^P < 0.05, compared with the model group at the same time-points.

In the open field test, the horizontal movement of rats in the model group decreased on day 7 and 14 compared to the sham group (P < 0.05), whereas the rats in MI + BFG group showed a greater horizontal movement than model group (P < 0.05). No difference was found in the vertical movement among groups. In the elevated plus-maze test, rats in model group spent significantly lower time in the open arms than rats in the sham group at all timepoints (P < 0.05). Rats administrated with BFG spent more time in the open arms on day 7 and 14 when compared to the model group (P < 0.05). The three groups showed no difference in their entries into the open arms (Fig. 6C).

Level of GAD in hippocampal CA3 region in the model group decreased over time after the ligation surgery when compared to the sham group on day 7 and 14 (P < 0.05). These changes were reversed in the MI + BFG group where GAD level was higher than that in model group on day 7 and 14 (P < 0.05) (Fig. 6A and B).

Real time RT-PCR analysis of samples from hippocampal region showed that level of NMDAR1 and GABARA mRNA was lower in the model group compared to the sham group on day 14 (P < 0.05). However, after BFG intervention, the level of GABARA mRNA was increased on day 14 (P < 0.05) (Fig. 6D).

Level of adrenaline in peripheral blood in the model group peaked on day 7, slightly decreased on day 14 and was statistically higher than that in the sham group on day 7 and 14 days (P < 0.05). Importantly, level of adrenaline in peripheral blood was significant lower in rats administered with BFG when compared to the model group on day 7 and 14 (P < 0.05) (Fig. 6E).

### BFG promoted mobilization of BM-MSCs *in vitro*

**Figure 7:**
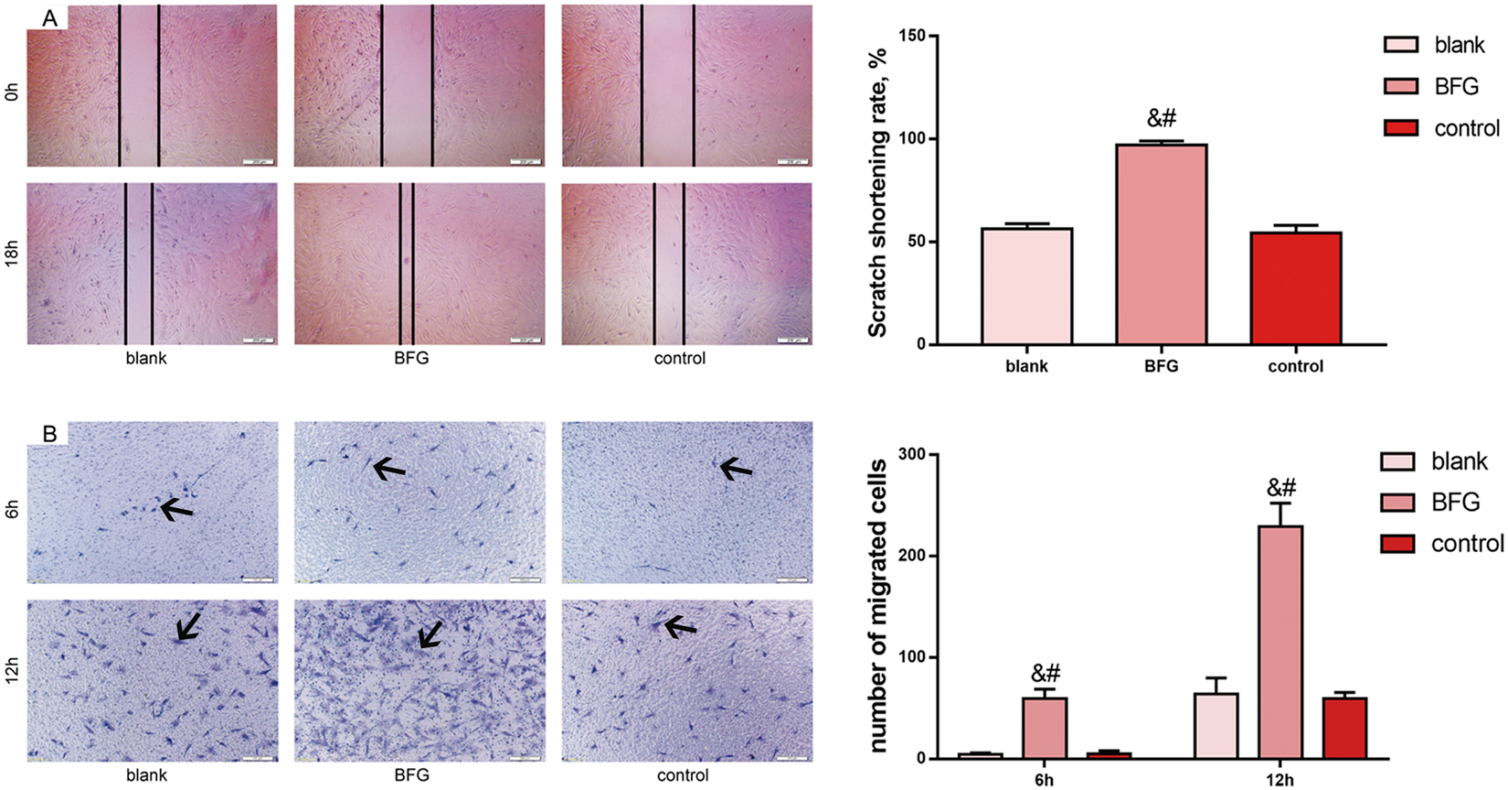
Migration of bone marrow mesenchymal stem cells *in vitro*. (**A**) Tablet scratch assay (×40 magnification); (**B**) Transwell assay (×100 magnification). Values are expressed as mean ± SD. BFG: Chaihulonggumulitang (*Bupleurum falcatum plus Dragon Bone and Oyster Shell Granules*). Complete medium: low-glucose Dulbecco’s modified Eagle’s medium supplemented with 15% (v/v) fetal bovine serum, 100 U/ml penicillin, and 100 μg/ml streptomycin. Blank group: BMSCs cultured with complete medium. BFG group: BMSCs dealt with complete medium containing 5% BFG serum of rats. Control group: BMSCs dealt with complete medium containing 5% normal serum of rats. ^&^P < 0.05, compared with the blank group; ^#^P < 0.05, compared with the control group.

Results of the *in vitro* tablet scratch experiment showed that cells treated with BFG serum showed a greater cell migration into a cell-free area than cells in other groups (P < 0.05) (Fig. 7A). In addition, more cells treated with BGF serum migrated from the upper chamber to the lower surface of the membrane (cells stained by crystal violet) than cells in control and blank group at 6 h and 12 h (P < 0.05) (Fig. 7B).

## Discussion

Our study demonstrated that in the setting of AMI, classical Chinese prescription BFG inhibited inflammatory responses, promoted BM-MSCs mobilization, improved heart damage and anxiety developed from AMI.

Traditional Chinese medicine has a history extending back to 2500 years. Symptoms of AMI could be categorized as *true heart pain* and *thoracic obstruction* while those of anxiety to *depressive*^16^. The interaction of blood stasis and phlegm-turbid is regarded as the main pathological changes in AMI complicated anxiety according to traditional Chinese medicine theory^15^. BFG is a classical Chinese prescription that promotes blood circulation to remove blood stasis, resolves phlegm and tranquilizes the mind^19^. BGF protects cardiomyocytes, inhibits platelet aggregation, and spontaneous activity in mice^22^.

BM-MSCs express surface molecules CD90, CD105 and adhesion-related antigens (CD54 and CD106). The neural and humoral signals produced by myocardial infarction activate BM-MSCs to egress from bone marrow^10^. The higher percentage of CD90 and CD105 positive cells in bone marrow and peripheral blood in our study confirmed that MI induced rapid mobilization of BM-MSCs. Migration is essential for stem cells to invade an ischemic tissue. In bone marrow, homing is a multistep process that shares components with the infiltration of leukocytes at inflammatory sites and has a significant role in interactions between stem cells and bone marrow endothelium^23^. BM-MSCs adhere and integrate into the endothelium to exit the blood circulation after being released from bone marrow to peripheral blood. Then, BM-MSCs transmigrate the endothelial barrier, penetrate the basement membrane and invade the surrounding tissue via the formation of plasmic podia^24^. The transmigration of BM-MSCs requires CD106 (vascular adhesion molecule-1, VCAM-1) expression on BM-MSCs and cardiac microvascular endothelium^24,25^. Similarly, CD54 (intercellular cell adhesion molecule-1, ICAM-1) regulates the migration of BM-MSCs to injured tissues^26^. In our study, percentage of CD54^+^ and CD106^+^ cells declined in bone marrow on day 3 after ligation surgery in the model group. The higher concentration of adhesion factors at the initial stage induced aggregation and infiltration of leukocyte to promote an inflammatory reaction. As their concentration dropped, adhesion between BM-MSCs and marrow stroma decreased, and BM-MSCs were released into the blood. This has corresponded with the higher percentage of CD90 and CD105 in peripheral blood and bone marrow on day 7. Interestingly, levels of adhesion factor in peripheral blood were elevated and peaked on day 7 in the model group which indicated a stronger adhesion of BM-MSCs to endothelium. When the contents decreased on day 14, more BM-MSCs exited blood circulation and settled in heart. Stem cell factor was also measured because it has been shown to promote mobilization and differentiation of BM-MSCs^27,28^. In the whole process, BFG increased content of stem cell factor in the peripheral blood, mediated adhesion and enhanced mobilization of BM-MSCs.

In addition, our results showed the CD105 expression in the marginal zone of myocardial infarction was enhanced by BFG. CD105 is a major glycoprotein of vascular endothelium and can be used as the marker of angiogenesis or BM-MSCs^29^. Based on the increased BM-MSCs in bone marrow and peripheral blood, we suggest that more BM-MSCs were recruited to the marginal zone of myocardial infraction by BFG administration, thus causing a higher expression of CD105. But it may also be interpreted that BGF promoted neovascularization after AMI. Therefore, the evidence is not conclusive and further research is needed to delineate these mechanisms.

Heart damage caused by myocardial infarction is a result of ventricular remodeling, pathological changes such as myocardial thinning and elongation of ventricular wall in infarction^30,31^. Our results demonstrated that rats suffering from heart failure had a lower LVEF and LVFS, thinner walls and increased ventricular diameter and volume, which worsened over time. BFG significantly prevented those pathological changes.

The dramatic cardiomyocyte death in myocardial infarction initiates an inflammatory response in AMI^30^. Activation of inflammatory cells induce endothelial dysfunction in coronary and initiate ventricular remodeling^2,30^. Nuclear transcription factor κB (NF-κB) and its downstream proinflammatory cytokine tumor necrosis factor-α (TNF-α) are responsible for inflammatory cascades after AMI^30^. BM-MSCs reduce the local inflammatory response by altering function of T cells, B cells, dendritic cells, and NK cells^9,32^. We showed a higher peripheral blood content of NF-κB and TNF-α in the sham group and the model group on day 3. The values in the sham group gradually decreased over time while those in the model group remained high. It is possible that higher levels of NF-κB and TNF-α in the sham group on day 3 were induced by injury of skin and muscle during the surgery, which recovered gradually. Whereas, inflammation caused by ligation surgery persisted and thus exhibited higher levels of NF-κB and TNF-α in peripheral blood in the model group. Our results demonstrated that BFG intervention inhibited inflammation, which may partly attribute to the higher mobilization rate of BM-MSCs.

It is plausible that symptoms of anxiety following AMI are product of systemic inflammation^33,34^. The behavioral symptoms of anxiety disorders are associated with alterations in GABAergic transmission in brain^35^. The excessive excitatory neurotransmitter glutamate and insufficient inhibitory neurotransmitter GABA over-activate the central nervous system and results in anxiety. The 67KDa isoform of glutamic acid decarboxylase (GAD67), which regulates GABA synthesis from glutamate, plays a particularly important role in inhibitory synaptic transmission. As a primary brain region of limbic system, hippocampus has been extensively studied in anxiety disorders^36^. Reduced GAD67 and GABA in hippocampus initiate anxiety-like behavior^37^. Glutamate receptor N-methyl-D-aspartate receptor (NMDAR) and γ-aminobutyric acid A receptor (GABAAR) are the target site of anxiolytics^38,39^. In addition, anxiety-like behavior is accompanied by enhanced sympathetic nerve activity, presenting a higher level of adrenaline^40^. The open-field test and elevated plus-maze test are classical methods to assess anxiety-like behavior. Rats suffering from anxiety may display decreased locomotor activity in the open-field test and an inclination to stay in the closed arms of elevated plus-maze test^41,42^. Our results showed that rats in the model group developed anxiety-like behavior with higher level of adrenaline in peripheral blood. In addition, GAD level decreased while the expression of NMDAR mRNA and GABAAR mRNA were downregulated after AMI. These changes after AMI were significantly improved by administration of BFG.

To concluded, our data showed that BFG alleviated inflammatory response in myocardial infarction, promoted the mobilization of BM-MSCs *in vivo and vitro*, attenuated heart damage in myocardial infarction, and improved anxiety behavior developing from myocardial infarction. Inflammation appears to be a common denominator in these outcomes as BM-MSCs have systemic anti-inflammatory activities^32^, inflammatory response caused by injured myocardium contribute to the heart damage^30^, and symptoms of anxiety following AMI may be a result of systemic inflammation^34^. In light of these observations, we suggested that enhanced mobilization of BM-MSCs by BFG may contribute to the anti-inflammatory effects, thus leading to improvement of heart damage and anxiety in myocardial infarction.

Our study has several limitations. First, we can’t be very sure that the anti-inflammatory effect of BFG was due to BM-MSCs mobilization and further research was needed to confirm it. Second, although CD90 and CD105 are surface markers of BM-MSCS, they also are expressed in other tissues. Thus, it might be inappropriate to say that these mobilized cells in the *in vivo* study are BM-MSCs for certain. However, the tablet scratch assay and Transwell assay in the *in vitro* study showed that mobilization of BM-MSCs were enhanced after BGF intervention, which provided as a strong evidence that the mobilized cells in the *in vivo* study might be BM-MSCs.

Overall, our findings uncover the underlying mechanisms of protective effects of BFG in psycho-cardiology outcomes downstream of AMI and suggest that BFG may be used as a therapeutic intervention in AMI to improve psycho-cardiology effects.
